# Supplementary material for: MYC is sufficient to generate mid-life high-grade serous ovarian and uterine serous carcinomas in a p53-R270H mouse model
Source: bioRxiv. 2024 Jan 29:2024.01.24.576924. Preprint. [Version 1] doi: 10.1101/2024.01.24.576924 (PMC10862747; doi:10.1101/2024.01.24.576924)
Supplement: Supplement 1 [file media-1.pdf]

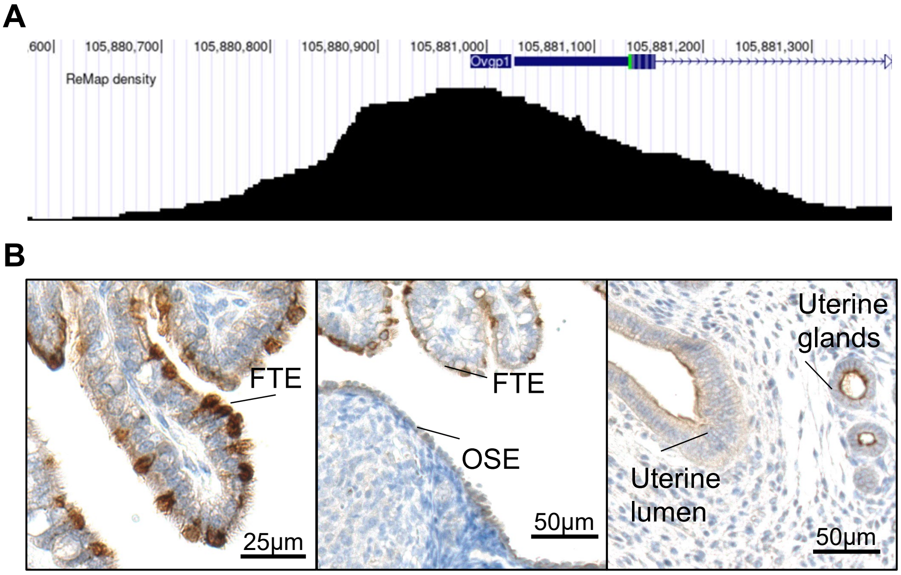

Figure S1: *Ovgp1* transgene expression. (A) UCSC Genome Browser display of the murine *Ovgp1* promoter with its regulatory peak, as annotated in the ReMap ChIP-seq database. (B) 3xHA tag transgene immunohistochemical staining at 10 weeks of age in an OvTrpMyc female.

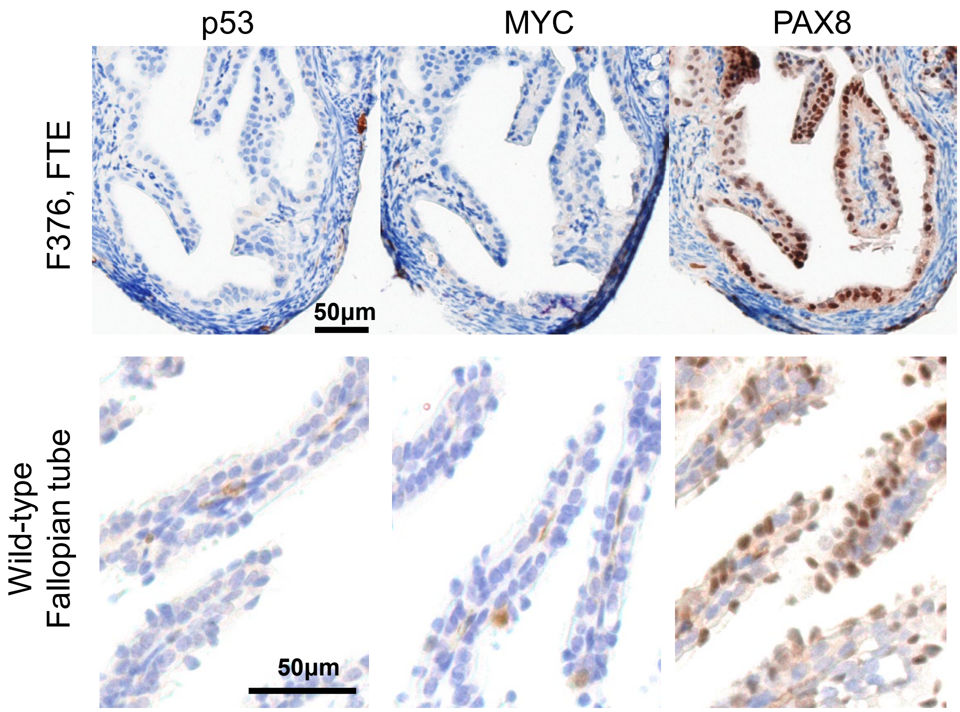

Figure S2: Absence of p53 and MYC staining in mouse with uterine epithelial expression of p53 and MYC. Staining of the FTE of OvTrpMyc mouse F376, associated with Fig. 5. Lower panel compares to wild-type mouse staining, at 12-months of age

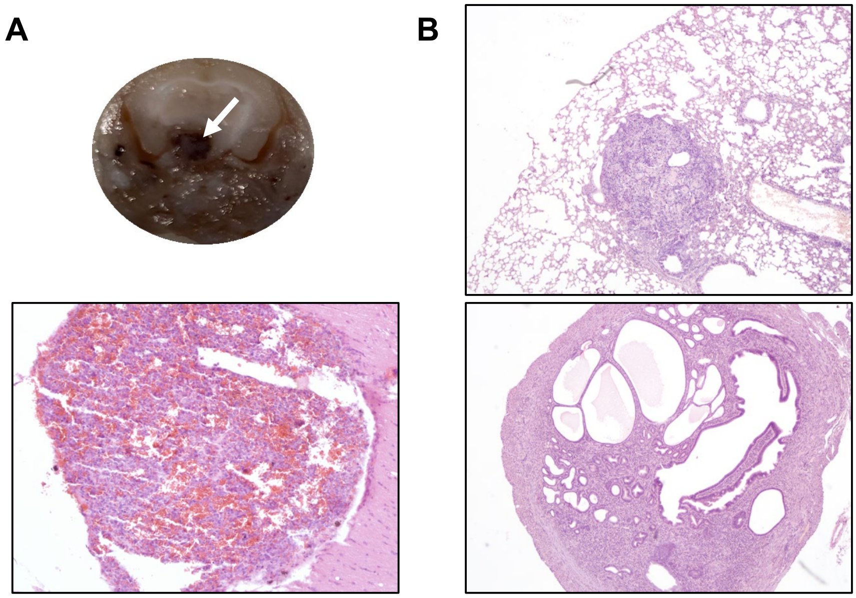

Figure S3: Necropsy of mice lacking macroscopic disease. OvTrpMyc mouse were evaluated by necropsy when macroscopic tumors were not immediately observed. **(A)** Dissected brain (top panel) and H&E section of brain tumor (bottom panel). **(B)** Lung metastasis (top panel) and a normal phenotype of some aged mice: cystic uterus (bottom panel).

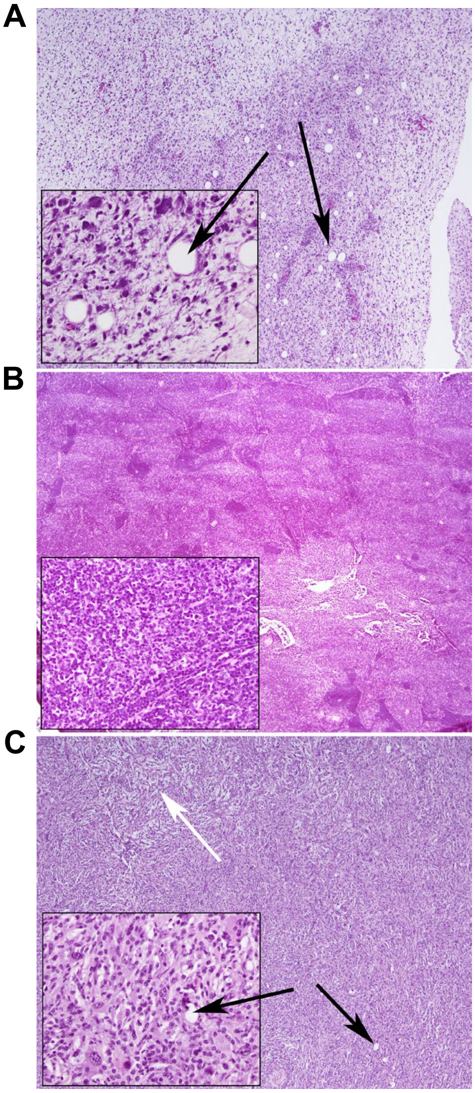

Figure S4: Other malignancies. (**A**) Myxoid neoplasm with markedly pleomorphic cells with associated vacuoles, marked by the black arrows. (**B**) Small round blue cell malignancy with relatively uniform nuclei growing in a sheeted growth pattern. (**C**) Tumor characteristics include spindle cell malignancy with scattered giant cells, storiform architecture, marked by the white arrow. Moderately pleomorphic with scattered vacuoles, marked by black arrows.
